# Phase transitions in rutile-related V_0.92_O_2_ synthesized at high pressures and tem­per­a­tures

**DOI:** 10.1107/S2052252525010693

**Published:** 2026-01-01

**Authors:** Andrzej Grzechnik, Václav Petříček, Pascal Reiss, Paul Zakalek, Dmitry Chernyshov, Karen Friese

**Affiliations:** a Jülich Centre for Neutron Science, Research Center Jülich, Jülich, 52425, Germany; b Institute of Crystallography, RWTH Aachen University, Aachen, 52066, Germany; c Institute of Physics, Academy of Sciences of the Czech Republic, Praha, 162 00, Czech Republic; d Quantum Materials Department, Max Planck Institute for Solid State Research, Stuttgart, 70569, Germany; e Swiss-Norwegian Beamlines, European Synchrotron Radiation Facility, Grenoble, 38043, France; Tsinghua University, China

**Keywords:** vanadium oxides, extreme conditions, phase transitions, structure–property relationships

## Abstract

Structural phase transitions in metastable rutile-related V_0.92_O_2_ synthesized at 10 GPa and 1273 K were studied with single-crystal X-ray diffraction in the tem­per­a­ture range 110–500 K and at pressures up to 9.2 GPa. V_0.92_O_2_ starts to decom­pose at 470 K and atmospheric pressure. When heated to tem­per­a­tures above about 350 K, the material transforms to an incommensurate phase. The oxygen sublattice is essentially rigid and it is mostly the V atoms that are affected by the modulation. An anharmonic description of the displacement parameters and their corresponding modulation is used for the V atoms to reach satisfactory agreement factors for main and first-order satellite reflections, indicating substantial disorder in the modulated structure. Measurements of electronic transport properties provide evidence that the incommensurate phase is insulating. On com­pression at room tem­per­a­ture, V_0.92_O_2_ reaches the ideal rutile structure at about 5.0 GPa. Both structural and electronic phase transitions are of the first-order character. The results of this work demonstrate that the structural and electronic behaviour of V_0.92_O_2_ at extreme conditions is distinctly different from that of stoichiometric VO_2_.

## Abstract 2

Phase transitions in metastable rutile-related V_0.92_O_2_ were studied with single-crystal X-ray diffraction and measurements of electronic transport properties at extreme conditions. The high-tem­per­a­ture incommensurate phase was found to be insulating.

## 1. Introduction

Vanadium dioxide (VO_2_) undergoes a first-order metal–insulator phase transition (MIT) from the rutile (phase *R*, *P*4_2_/*mnm*, *Z* = 2) to the monoclinic (phase *M*1, *P*2_1_/*c*, *Z* = 4) polymorphs at about *T*_MIT_ = 341 K (Qazilbash *et al.*, 2007 ▸; Shao *et al.*, 2018 ▸; Liu *et al.*, 2020 ▸; Pouget, 2021 ▸; Xue & Yin, 2022 ▸; Joshi *et al.*, 2023 ▸). The metallic *R* phase is built of chains of edge-sharing VO_6_ octa­hedra connected by common corners. The V—V distance of 2.85 Å along the chains is equal to the *c* lattice parameter. The structure of the insulating *M*1 phase is also made of two VO_6_ chains, but with V–V dimers in a zigzag pattern due to displacement of the V atoms from the ideal rutile positions (Longo *et al.*, 1970 ▸). The *R*→*M*1 structural instability gives rise to pre-transitional diffuse scattering (Pouget, 2021 ▸). MIT, which is an improper ferroelastic phase transition, is associated with a magnetic susceptibility drop (Pouget, 2021 ▸) and a change in thermochromic properties (Mamakhel *et al.*, 2022 ▸).

Doping with low-valence cations (*e.g.* Al^3+^, Cr^3+^ and Fe^3+^) stabilizes two additional insulating polymorphs, which are monoclinic (phase *M*2, *C*2/*m*, *Z* = 8) and triclinic (phase *T*, *P*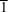, *Z* = 4). In *M*2, there are alternate short and long distances in the linear V—V chain, while the V atoms are equidistant in the other zigzag V—V chain (Marezio *et al.*, 1972 ▸; Ghedira *et al.*, 1977 ▸). The distortion in *T* with respect to *M*2 is mainly due to breaking of the linearity and pairing of the V atoms in the zigzag chain (Ghedira *et al.*, 1977 ▸). The stabilities of phases *R*, *M*1, *M*2 and *T* are affected by stoichiometry, electric field, strain or pressure (Marezio *et al.*, 1972 ▸, Ghedira *et al.*, 1977 ▸; Atkin *et al.*, 2012 ▸; Liu *et al.*, 2018 ▸; Pouget, 2021 ▸; Wilson *et al.*, 2022 ▸; Bouvier *et al.*, 2023 ▸; Joshi *et al.*, 2023 ▸). *M*1 and *M*2 can transform into one another, with *T* as an inter­mediate (Marezio *et al.*, 1972 ▸). *M*2 could also be an inter­mediate in the *M*1→*R* transformation (Liu *et al.*, 2020 ▸; Bleu *et al.*, 2023 ▸). The three insulating phases may co-exist and form domains (Lu *et al.*, 2014 ▸). The high-valence dopants (*e.g.* Nb^5+^, Mo^6+^ and W^6+^) lower the *T*_MIT_, while the low-valence dopants (*e.g.* Al^3+^, Cr^3+^ and Fe^3+^) increase it (Pouget, 2021 ▸; Xue & Yin, 2022 ▸; Joshi *et al.*, 2023 ▸). In the first case, the charge is com­pensated by the presence of the V^3+^ cations. In the latter, it is com­pensated by the V^5+^ cations.

^51^V NMR studies of the metallic and insulating phases of VO_2_ provide contradictory results. Boyarsky *et al.* (2000 ▸) suggested that MIT is accom­panied by the change of the electronic state 2V^4+^ ↔ V^3+^ + V^5+^, with the presence of two structurally and chemically different vanadium cations V^3+^ and V^5+^ in *M*1. On the other hand, the data by Gro Nielsen *et al.* (2002 ▸) show the presence of only V^4+^ cations in the insulating phase. Electrical and photoemission investigations by Joshi *et al.* (2023 ▸) demonstrated that the V^3+^ and V^5+^ cations co-exist in the metallic state of stoichiometric VO_2_ due to charge fluctuations of the V^4+^ cations. The insulating phase is sup­pressed in VO_2–*x*_, while the metallic phase is suppressed in VO_2+*y*_. However, Joshi *et al.* (2023 ▸) did not present structural data for any of the VO_2–*x*_ and VO_2+*y*_ phases across MIT.

The pressure–tem­per­a­ture phase diagram of VO_2_ was determined by Chen *et al.* (2017 ▸). In addition to the *R* and *M*1 polymorphs, one insulating *M*1′ (monoclinic) phase and two metallic phases *X* (triclinic) and *O* (ortho­rhom­bic) were identified. When *M*1 is com­pressed at room tem­per­a­ture, it transforms to *M*1′ at 13.9 GPa (Bouvier *et al.*, 2023 ▸). *X* co-exists with *M*1′ in the pressure range 32–42 GPa. Upon decom­pression, *X* transforms to another (unidentified) phase between 20 and 3 GPa. The sequence of phase transitions in VO_2_ under strong com­pression was examined by Xie *et al.* (2018 ▸).

Apart from VO_2_, the experimentally determined equilibrium phases in the central part of the V–O phase diagram (VO_*x*_, 1.5 ≤ *x* ≤ 2.5) at atmospheric pressure are V_2_O_3_, V_*n*_O_2*n*–1_ (*n* = 3 ÷ 8), V_3_O_7_, V_6_O_13_ and V_2_O_5_ (Wriedt, 1989 ▸). The calculated phase diagram for 1.5 ≤ *x* ≤ 2.5 includes only V_2_O_3_, V_3_O_5_, VO_2_, V_3_O_7_ and V_2_O_5_ (Hu *et al.*, 2023 ▸). The oxides V_*n*_O_2*n*–1_ (*n* = 3 ÷ 9) form a Magnéli homologous series (Schwingenschlögl & Eyert, 2004 ▸; Allred & Cava, 2013 ▸) according to the formula V_*n*_O_2*n*–1_ = V_2_O_3_ + (*n* − 2)VO_2_. With respect to the com­position of vanadium dioxide, they are anion deficient and can also be expressed as VO_2–*y*_. The end members of this series are corundum V_2_O_3_ (*R*
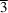
*c*, *Z* = 6) and rutile VO_2_ (phase *R*), both with a hexa­gonal close-packed array of O atoms (Katzke *et al.*, 2003 ▸). One oxygen layer is removed at every *n*th vanadium layer in the direction perpendicular to the (211) plane of the parent rutile structure. Magnéli phases are also known for the titanium, niobium and tungsten oxides (Wu *et al.*, 2023 ▸).

The homologous series of vanadium oxides V_*n*_O_2*n*+1_ in the V_2_O_5_–VO_2_ system was predicted by Wadsley (1957 ▸). Its formula can be written as V_*n*_O_2*n*+1_ = V_2_O_5_ + (*n* − 2)VO_2_ for 2 ≤ *n*. With respect to the chemical com­position of VO_2_, the Wadsley oxides are cation deficient, *i.e.* V_1–*x*_O_2_. The end members of this series are α-V_2_O_5_ and VO_2_(B) (Katzke *et al.*, 2003 ▸). These materials have structures derived from VO_*x*_ (*Fm*
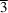
*m*, *Z* = 4), where *x* ≃ 1, by introducing different ordered vacancies in the oxygen cubic close-packing array. Shear deformations break symmetry and cause the collapse of the face-centred cubic layers along the cubic *c* axis. The cations partially fill out the available voids in the oxygen sublattice in a commensurate way.

Metal-deficient V_1–*x*_O_2_ material, synthesized at 6.5 GPa and 1273 K by substituting V^5+^ into VO_2_ (Chamberland, 1973 ▸), has a distorted rutile structure (*P*2/*m*, *Z* = 2), with the V—V distances in both chains equal to the *b* lattice parameter (Galy & Miehe, 1999 ▸). Up to 10 wt% of V_2_O_5_ was accommodated in V_1–*x*_O_2_ at these conditions. Based on the resistivity data, the tem­per­a­tures for MIT depend on the actual com­position (Chamberland, 1973 ▸). They increase from 353 to 361 K for 2 wt% (V_0.995_O_2_) and 10 wt% of V_2_O_5_ (V_0.976_O_2_), respectively. No structural details of the metallic phase were provided.

**Figure 1:**
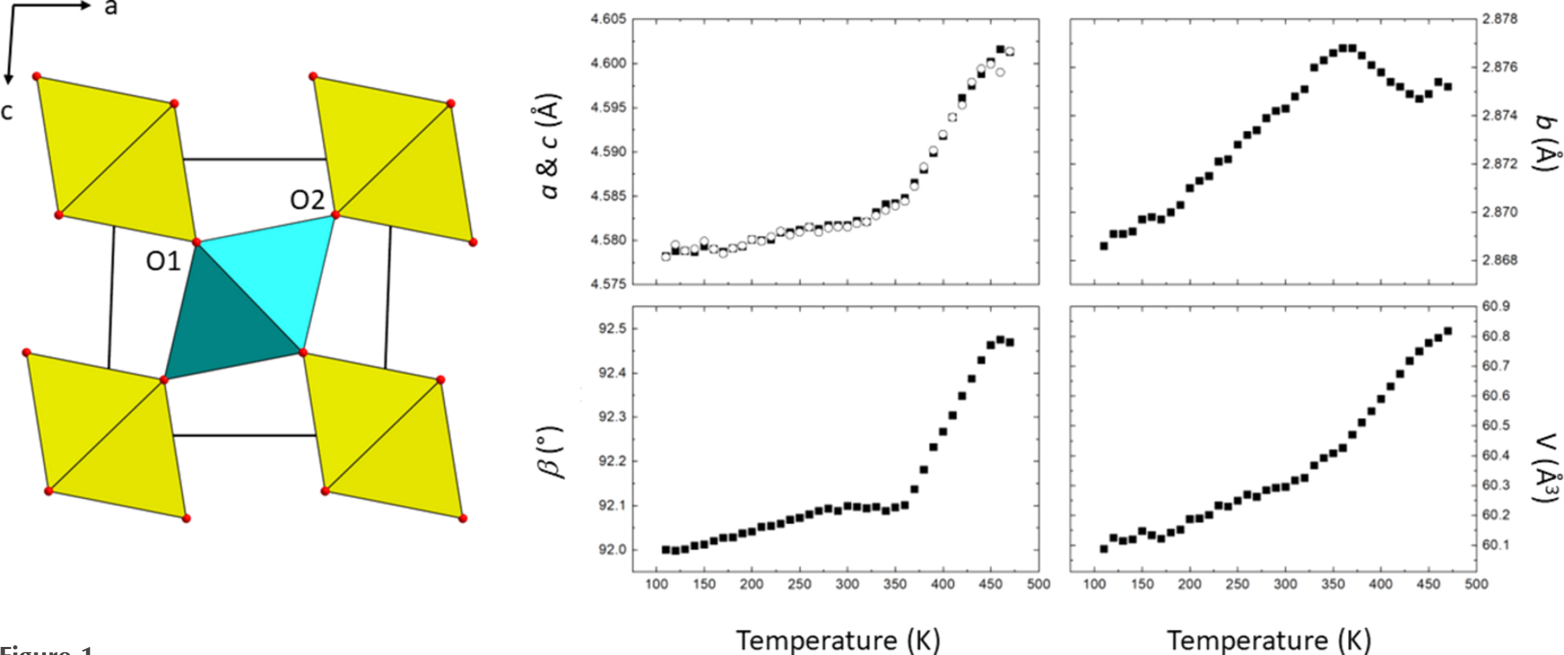
The average crystal structure in the space group *P*2/*m* (*Z* = 2), as well as the tem­per­a­ture dependence of its lattice parameters and unit-cell volumes. The octa­hedra around the V1 and V2 atoms are drawn in yellow and cyan, respectively. The O1 and O2 atoms are labelled. The symbols in the plots of the lattice parameters and unit-cell volumes are larger than the estimated standard deviations.

Recently, we have grown single crystals of rutile-related V_0.92_O_2_ at 10 GPa and 1273 K from a polycrystalline starting material of the Wadsley phase V_6_O_13_ (Grzechnik *et al.*, 2024 ▸) corresponding in terms of its com­position to V_1–*x*_O_2_ (Chamberland, 1973 ▸) or VO_2+*y*_ (Joshi *et al.*, 2023 ▸) with 35 wt% of V_2_O_5_. *In situ* synchrotron measurements revealed that this new phase forms above 500 K in the pressure range 4–17.5 GPa and can be recovered to ambient conditions. The characteristic feature of its crystal structure (*C*2/*m*, *Z* = 4) is the presence of disorder affecting the V atoms, which occupy the two split-atom positions V1 and V2. The V1 atoms in one of the octa­hedral chains are displaced along the *b* axis, while the V2 atoms in the other are fourfold split in the *bc* plane. This results in two zigzag V—V chains, one with equidistant V1 atoms and the other with short and long V2—V2 distances. Disregarding the split V-atom positions, the average structure (*P*2/*m*, *Z* = 2) of this new phase (Fig. 1 ▸) is like that for the V_1–*x*_O_2_ material (Chamberland, 1973 ▸; Galy & Miehe, 1999 ▸). Pseudosymmetry considerations (Grzechnik *et al.*, 2024 ▸) indicate that it is the ordered variant of *M*2. The transformation of the Wadsley phase V_6_O_13_ into rutile-related V_0.92_O_2_ involves the transition from cubic to hexa­gonal close-packing of the O atoms.

The physical properties of different structural forms of V_1–*x*_O_2_ are closely correlated with the formation and spatial arrangement of the short V—V distances (Liu *et al.*, 2018 ▸; Joshi *et al.*, 2023 ▸). Detailed experimental characterization of new types of structural ordering, such as the (in)commensurate long- or short-range order, are a necessary step towards the better understanding of structure–property relationships and the further control of the physical properties of vanadium oxides. While browsing the literature on (non)stoichiometric vanadium dioxides, one can readily see that most of the articles are somewhat incom­plete in the sense that either the structural or electronic properties and transformations of VO_2±*x*_ are investigated. In this article, we combine both approaches (i) to determine whether metastable V_0.92_O_2_ undergoes any tem­per­a­ture- or pressure-induced phase transitions using single-crystal X-ray diffraction, and (ii) to examine its electronic transport properties at atmospheric pressure for further elucidation of the structure–property relationships in vanadium dioxides.

## 2. Experimental methods

Single-crystal growth of V_0.92_O_2_ from the Wadsley phase V_6_O_13_ was described previously (Grzechnik *et al.*, 2024 ▸).

Synchrotron single-crystal diffraction measurements (λ = 0.72044 Å) were performed on the BM01 station of the Swiss–Norwegian Beamlines (SNBL) at the European Synchrotron Radiation Facility (Grenoble, France) (Dyadkin *et al.*, 2016 ▸). The data (a full rotation of 360°) were collected using a Pilatus 2M detector. After several tests on various crystals at room tem­per­a­ture to check for their quality and for any radiation damage (Grzechnik *et al.*, 2023 ▸), our data collection strategy was to measure frames with a fine angular slicing of 0.1° and the exposure time of 0.1 s/frame. The chosen crystal was mounted on a glass pin and placed in the stream of nitro­gen from an Oxford Cryostream 700+. It was cooled to 100 K and the data were collected on heating to 500 K with a step of 10 K.

High-pressure single-crystal X-ray data at room tem­per­a­ture were measured on a STOE IPDS-II (Stoe & Cie GmbH, Darmstadt, Germany; λ = 0.71073 Å) equipped with an image plate, as well as on a 4-circle Huber diffractometer with an Ag microfocus Incoatec source (λ = 0.5608 Å) and a Pilatus 300k detector. A crystal of V_0.92_O_2_ was loaded into the Ahsbahs diamond anvil cell (Ahsbahs, 2012 ▸), together with a 4:1 (*v*/*v*) methanol–ethanol pressure medium and a ruby ball as a pressure marker (Mao *et al.*, 1986 ▸).

All the laboratory and synchrotron data collected with the Pilatus detectors were analysed with the program *CrysAlis PRO* (Rigaku Oxford Diffraction, 2024 ▸). The data from the IPDS-II diffractometer were processed with the program *X-AREA* (Stoe & Cie, 1998 ▸). Solution and refinement of the structures were carried out with the programs *JANA2006* (Petříček *et al.*, 2014 ▸) and *JANA2020* (Petříček *et al.*, 2023 ▸).

Electronic transport measurements were carried out between 250 and 400 K inside a Quantum Design PPMS system, employing the electrical transport option (ETO). We used a standard four-wire measurement technique with a low AC excitation current of 100 µA to avoid parasitic sample heating and a frequency around 57 Hz. Good electronic contacts were made by attaching 10 µm Au wires to a single crystal of approximate dimensions 0.15 mm × 0.10 mm × 0.08 mm using an Ag paint. We confirmed a negligible phase angle of less than 1° over the tem­per­a­ture range.

## 3. Results and discussion

### 3.1. Single-crystal X-ray diffraction in the tem­per­a­ture range 110–500 K and under ambient pressure

On heating from 110 K to about 460 K, all the main reflections in the synchrotron data are indexed and integrated with the primitive monoclinic lattice corresponding to the average structure (*P*2/*m*, *Z* = 2) determined previously (Galy & Miehe, 1999 ▸; Grzechnik *et al.*, 2024 ▸). Metastable V_0.92_O_2_ starts to collapse at above 460 K as the reflections become smeared out. Also, new additional reflections appear. Above 470 K, the observed reflections cannot be indexed as originating from a single phase, indicating decom­position of the V_0.92_O_2_ material.

From the abrupt changes of the lattice parameters and unit-cell volumes (Fig. 1 ▸), it is seen that the material undergoes a first-order iso-symmetrical *P*2/*m*→*P*2/*m* phase transition at about 350 K. The *b* lattice parameter of the high-tem­per­a­ture phase is smaller than that for the low-tem­per­a­ture phase. The drop in this lattice parameter at the phase transition is correlated with an abrupt shortening of the V—V distances in the octa­hedral chains. The *a* and *c* lattice parameters exhibit the same evolution in the entire tem­per­a­ture range studied here. The β angle, which is a measure of a monoclinic distortion of the tetra­gonal rutile structure, increases with elevated tem­per­a­ture and has a drastic change of slope at about 350 K. It is remarkable that the tem­per­a­ture of the phase transition observed in V_0.92_O_2_ is very similar to that observed in V_1–*x*_O_2_, depending on the com­positional variable *x* (Chamberland, 1973 ▸; Qazilbash *et al.*, 2007 ▸; Liu *et al.*, 2020 ▸; Pouget, 2021 ▸; Joshi *et al.*, 2023 ▸).

**Figure 2:**
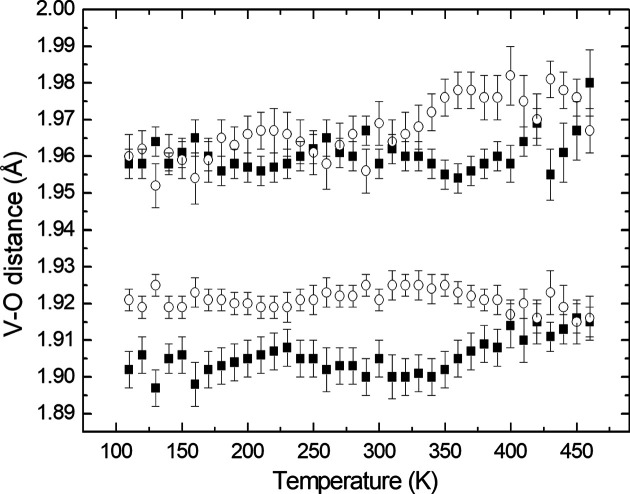
Temperature dependence of the V—O distances in the V1O_6_ (full symbols) and V2O_6_ (open symbols) octa­hedra in the average structure (*P*2/*m*, *Z* = 2).

**Figure 3:**
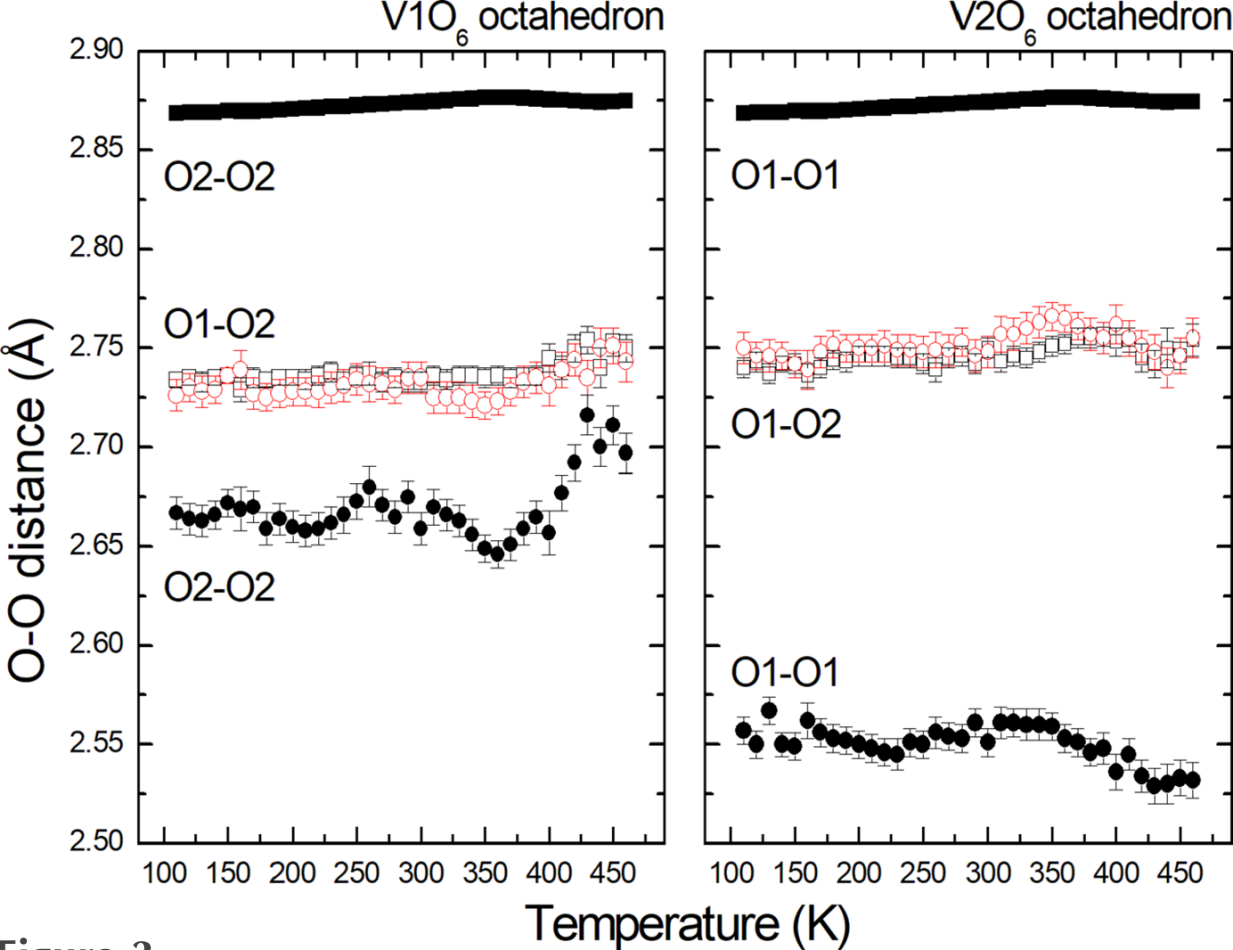
Temperature dependence of the O—O distances in the average structure (*P*2/*m*, *Z* = 2). The error bars are shown when larger than the symbols.

**Figure 4:**
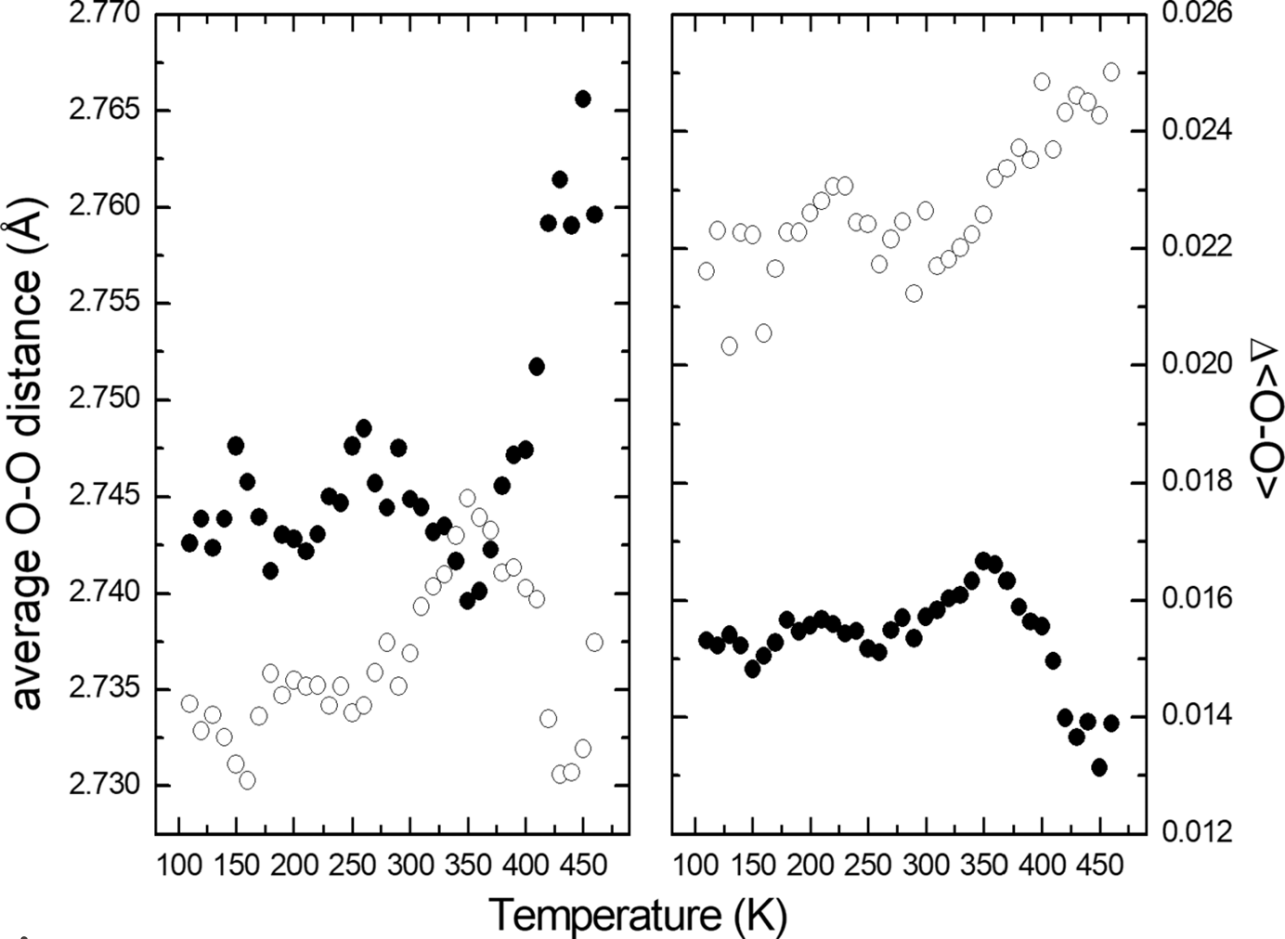
Average O—O distances and Δ〈O—O〉 deviation parameter in the V1O_6_ (full symbols) and V2O_6_ (open symbols) octa­hedra of the average structure (*P*2/*m*, *Z* = 2). The deviation parameter is defined as Δ〈O—O〉 = 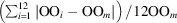.

The V—O distances in the average structure of V_0.92_O_2_(*P*2/*m*, *Z* = 2) as a function of tem­per­a­ture are shown in Fig. 2 ▸. There is no obvious anomaly that could be associated with the phase transition at about 350 K. However, the O—O distances in both chains of octa­hedra exhibit clear changes in their tem­per­a­ture dependencies at the phase transition (Fig. 3 ▸). The equatorial planes of the octa­hedra are defined by the atoms involved in edge sharing in the chains. The apical atoms in V1O_6_ and V2O_6_ are O1 and O2, respectively. The shortest and longest O—O distances are in the equatorial planes of both polyhedra, *i.e.* the O2—O2 distances in the V1O_6_ octa­hedra and the O1—O1 distances in the V2O_6_ octa­hedra. The shortest distances correspond to the shared edges of the octa­hedra. The most affected O—O distances at the phase transition are those in the respective equatorial planes. The average O—O distances abruptly change at the phase transition – they increase in V1O_6_, while they decrease in V2O_6_ (Fig. 4 ▸). The octa­hedron around the V1 atom at the site with the higher occupancy is less distorted than that around the V2 atom and becomes more regular above the phase transition.

**Figure 5:**
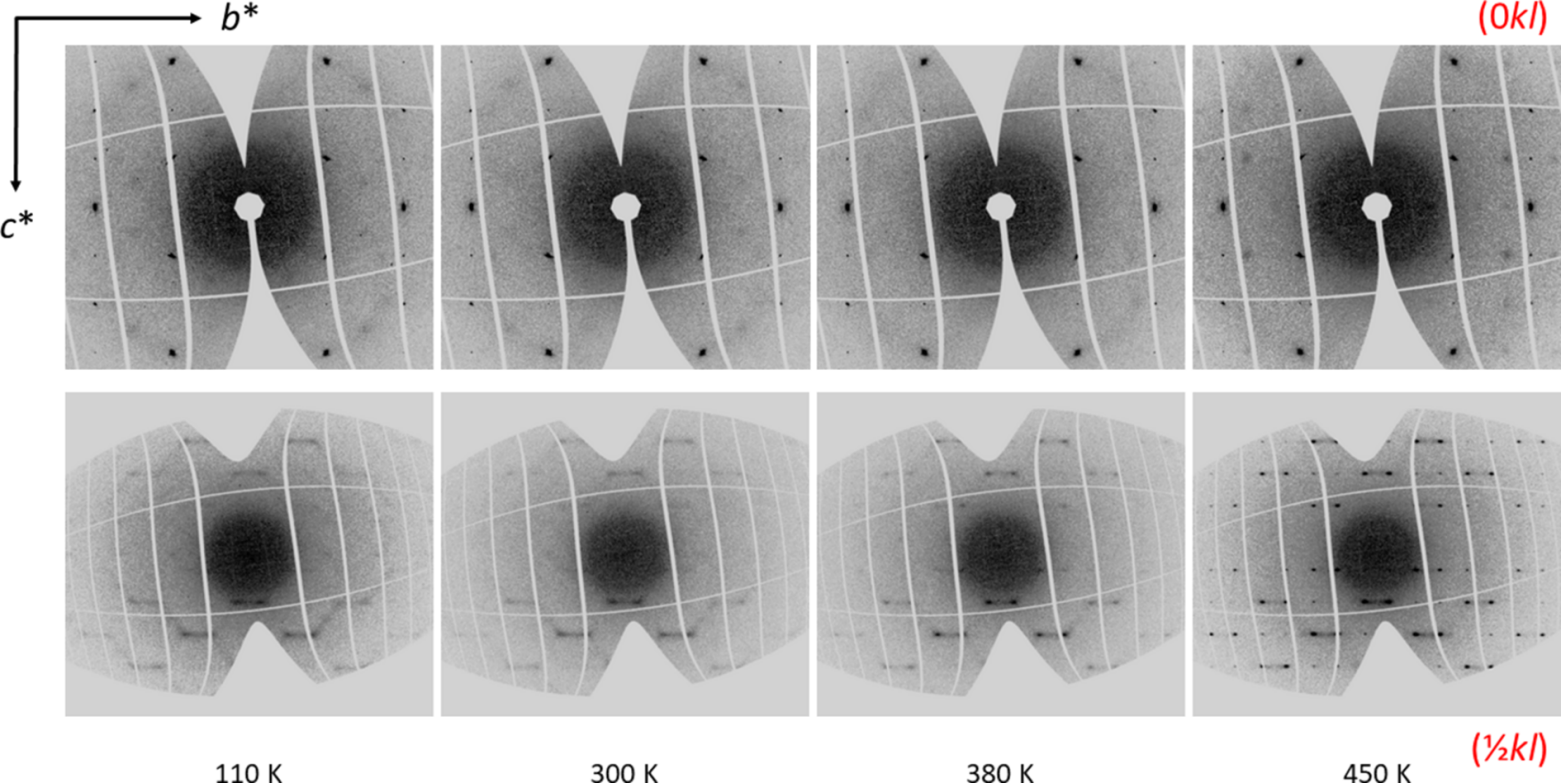
Reconstructions of the reciprocal space in the (0*kl*) and (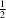
*kl*) planes (the upper and lower row, respectively) from the synchrotron data at selected tem­per­a­tures.

**Figure 7:**
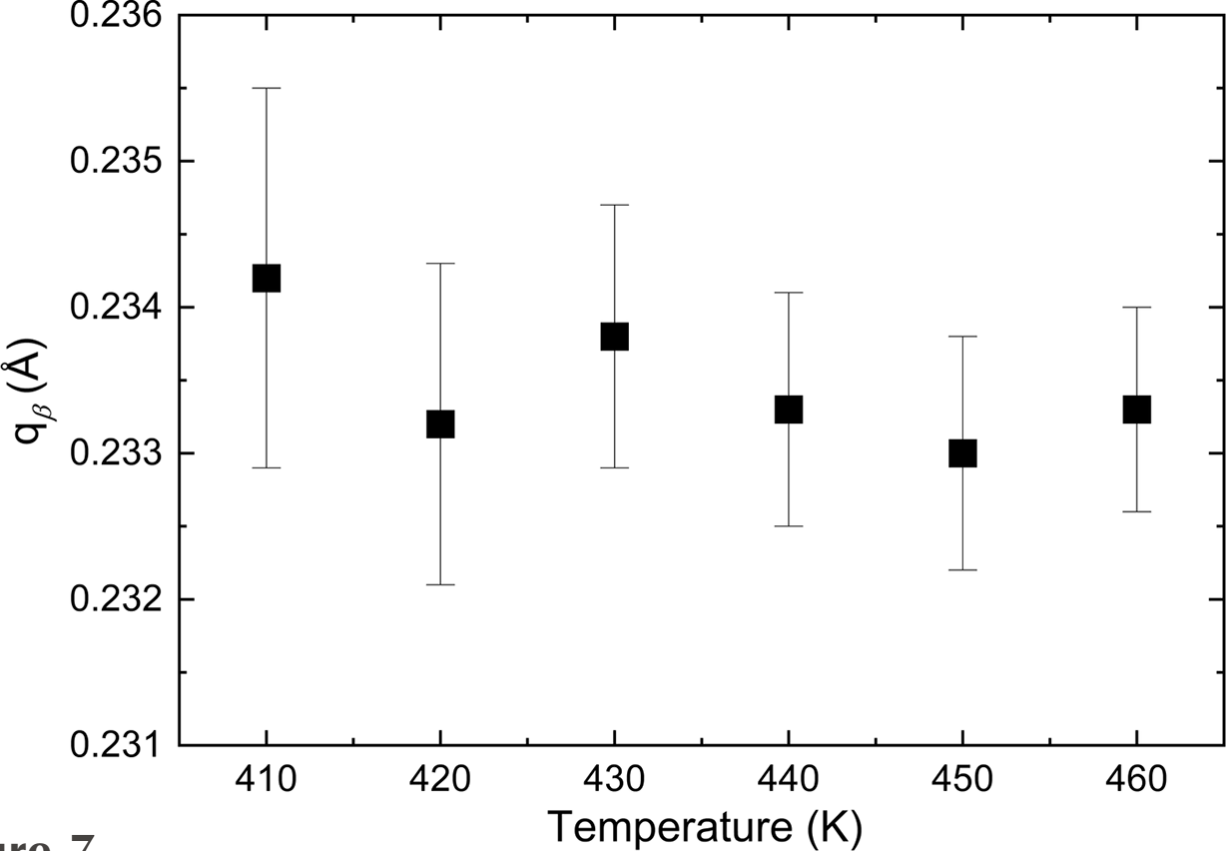
Temperature dependence of the *q*_β_ com­ponent of the incommensurate vector.

Diffuse scattering is not observed in the laboratory data measured either on the IPDS-II diffractometer (image plate, Mo *K*α radiation) or on the 4-circle diffractometer (Pilatus 300k detector, Ag microfocus source). However, the analysis of the reconstructions of the reciprocal space based on the synchrotron measurements reveals the presence of diffuse scattering at all tem­per­a­tures (Figs. 5 ▸ and 6 ▸). The intensity of the diffuse scattering increases close to the transition tem­per­a­ture. Above the phase transition, the diffuse features start to condense to well-defined satellite reflections. The strongest diffuse scattering is between the nearest satellites, which could be indexed with one incommensurate wave vector **q** = (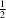, β, 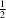), where β ≃ 0.234. This vector, which is essentially constant as a function of tem­per­a­ture, can only be precisely determined above 400 K (Fig. 7 ▸).^1^ All these observations indicate that metastable V_0.92_O_2_ does not transform to the ideal rutile structure (*R*) at high tem­per­a­tures and ambient pressure.

All the main and satellite reflections in the diffraction pattern at 460 K can be indexed and integrated with monoclinic lattice parameters *a* = 4.5995 (4), *b* = 2.8746 (2), *c* = 4.6014 (4) Å and β = 92.49 (1)° in combination with the vector **q** = [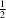, 0.2328 (9), 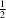]. Only first-order satellites were detected. Satellites of the second order do not show any significant intensity in the integration and are also not visible in the reconstructions of reciprocal space. For all the refinements, the overall stoichiometry and occupancies for V1 and V2 were fixed to the values reported by Grzechnik *et al.* (2024 ▸), as a free refinement of the occupation parameters showed only minor deviations from this stoichiometry.

The refinement of the average structure at 460 K was carried out in the space groups *P*2/*m*, *P*2, *Pm*, *P*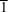 and *P*1. For the triclinic space groups, additional twinning *via* a twofold axis in the direction [010] was included. For none of the last four space groups were the overall agreement factors significantly better than for *P*2/*m*, considering the higher number of parameters in the refinements.

For the refinements of the incommensurate structure, the structure was transformed according to *a*′ = *a* + *c*, *b*′ = *b* and *c*′ = −*a* + *c*, with a resulting **q** vector of [0, 0.2328 (9), 0] to a pseudo-ortho­rhom­bic *X*-centred cell with *X* = (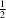, 0, 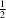, 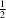) and an angle of 90.02° (Table 1 ▸). Several trial refinements were performed in monoclinic and triclinic superspace groups. Considering the agreement factors and the number of parameters in the refinement, the best result was obtained in superspace group *X*2/*m*(0β0)*s*0. Additional twinning with a twofold axis in the direction [001] was included. The introduction of the twinning led to a significant decrease in the agreement factors for the satellite reflections. In this model, initially only the first harmonics of the Fourier coefficients of a displacive modulation of the V and O atoms were considered. This led to an unsatisfactory *R*(obs) agreement factor of approximately 25% for the satellite reflections and high difference density in the difference Fourier map around the V atoms. When the second harmonic of the displacive modulation function for the V atoms was added, a substantial decrease in the overall agreement factors for the satellite and main reflections was achieved, while introducing higher harmonics for oxygen or an occupational modulation wave for vanadium did not result in better agreement factors (while leading to an increased data-to-parameter ratio). However, a trial calculation of the intensities of the second-order satellites showed that, assuming this model, their intensities would be substantial so that they should be clearly observed. As this is not the case, it is obvious that this model cannot be the correct one. Also, an inspection of the de Wolff sections and the refined modulation functions around the V-atom positions clearly showed a very bad agreement. We therefore discarded the model with higher harmonics of the displacive modulation.

Instead, we started from the assumption of a modulation of the displacement parameters. The electron density around the V atoms in the average structure (Fig. S1 in the supporting information) was best described using an anharmonic tensor. As the com­ponents of the third-order tensor for V are fixed to zero by the symmetry, we introduced a fourth-order anharmonic tensor in the average structure. This led to a substantial decrease in the agreement factors for the main reflections. Introducing a modulation of the anharmonic displacement parameters also led to a significant decrease of the agreement factor for the satellite reflections of first order, while the intensities of the second-order satellites were very small, in accordance with our observations.^2^

According to an earlier chemical analysis, the overall com­position of the com­pound is V_0.92_O_2_ (Grzechnik *et al.*, 2024 ▸), suggesting vacancies in the V sublattice. However, neither of the de Wolff sections showed any indication of a significant modulation of the height of maxima in the electron density, nor did the introduction and subsequent refinement of occupational modulation lead to better agreement factors. From these observations, we deduced that the V vacancies are randomly distributed within the V sublattice.

In the disordered room-tem­per­a­ture structure, the oxygen sublattice does not substanti­ally deviate from the ideal positions. This observation correlates very well with the fact that the O-atom positions and their displacement parameters are hardly affected by the modulation functions in the incommensurate structure (Table 2 ▸). Consequently, the O—O distances do not essentially vary as a function of the inter­nal parameter *t* if the standard deviations are considered (Table S1 in the supporting information). All these observations imply that the oxygen sublattice is nearly rigid (see animation S1 in the supporting information).

Amplitudes of the displacive modulation of the V atoms are also hardly significant and V—V distances are almost constant in the modulated structure (Table S2). We attribute the absence of significant displacements to the fact that the atoms are disordered over several split atom positions. In our structural model, the anharmonic displacement parameters and their corresponding modulations describe such a disorder of the atoms. While it is thus difficult to qu­antify the absolute displacements of the V atoms from their average positions, an inspection of the anharmonic displacement parameters reveals that the largest displacements are in the direction of *a* (animation S1). This is also clearly visible in animations S2 and S3, which show the joint probability density function (j.p.d.f.) around the V-atom positions in the modulated structure. Animations S4 and S5 show the j.p.d.f. around the O atoms. A complete animation of the incommensurate phase, including the j.p.d.f. for all the atoms in the unit cell, is in animation S6.

In the disordered room-tem­per­a­ture structure, the V1 atoms are arranged in a zigzag pattern, with a V1—V1 dis­tance of 2.9020 (2) Å. In the modulated structure, the average V1—V1 and V2—V2 distances are slightly smaller at 2.87489 (3) and 2.87484 (3) Å, respectively. Thus, surprisingly, the V—V distances are smaller at higher tem­per­a­tures. How­ever, the true positions of vanadium in the modulated structure are difficult to determine due to the additional disorder modelled by the anharmonic displacement parameters and their modulation.

Considering the V1O_6_ octa­hedra, in the modulated structures there are four shorter V—O bonds, plus two longer ones (Table S3). On the other hand, within the V2O_6_ octa­hedra, the situation is reversed and there are four longer and two shorter V—O bonds (Table S3).

In the disordered structure at room tem­per­a­ture, the bond valence sums (BVSs) of V1 and V2 are basically equal and amount to about 4.0 v.u. (with the BVS parameters for V^4+^) or about 4.4 v.u. (with the BVS parameters for V^5+^). In the modulated structure, the bond valence sums are smaller, with values of around 3.80 for both V atoms assuming a bond valence parameter of V^4+^ and 4.15 for a bond-valence parameter of V^5+^. Again, one must consider that these parameters are based on the average V-atom coordinates and do not really reflect the deviations from these average positions described by the anharmonic parameters.

### 3.2. Measurements of electronic transport properties at atmospheric pressure

**Figure 8:**
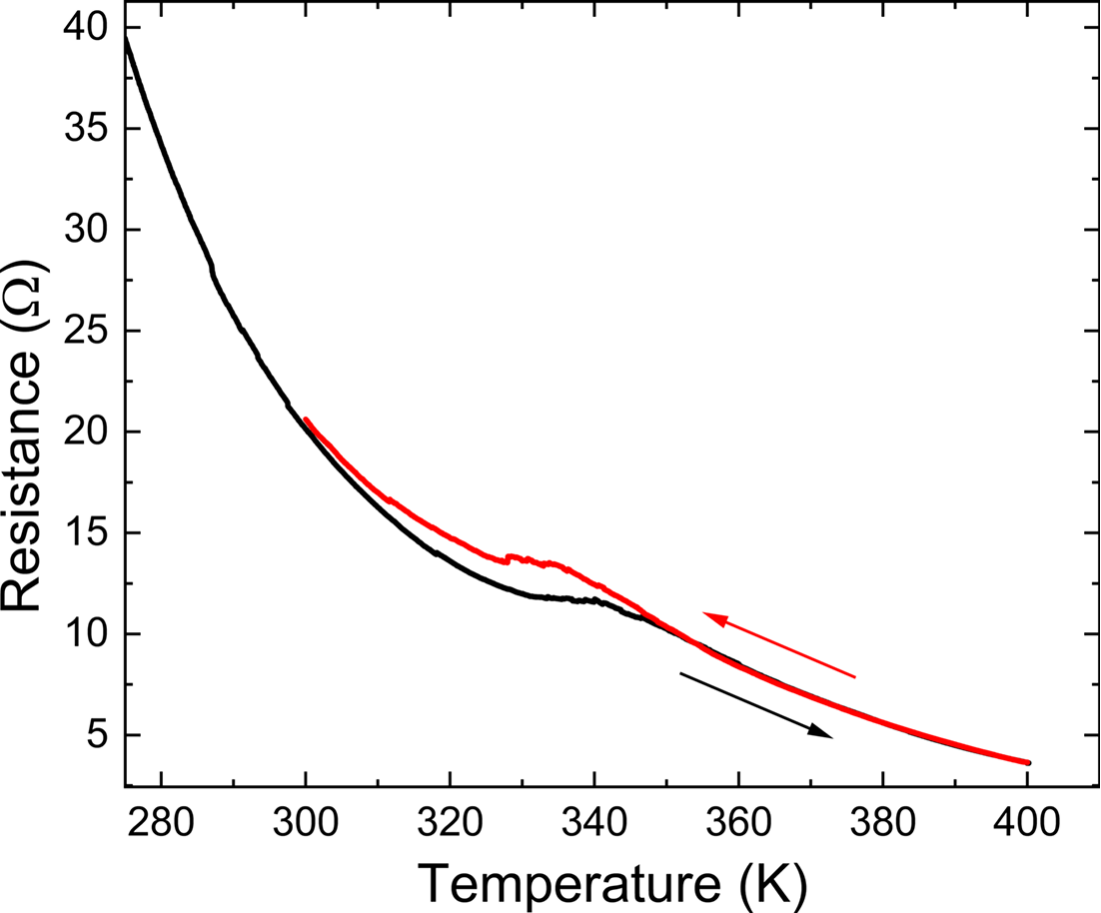
Resistance *versus* tem­per­a­ture on heating (black line) and cooling (red line) in the range 275–400 K.

Fig. 8 ▸ shows qualitatively the evolution of resistance measured on a single crystal of V_0.92_O_2_ on heating and cooling in the range 275–400 K. The onset of a first-order phase transition is at about 330 K. A hysteresis of about 10 K is observed on cooling the crystal from 400 K to room tem­per­a­ture. The data clearly show that both phases are non-metallic. V_0.92_O_2_ becomes even more insulating above the phase transition. Such a behaviour is different from that of (nearly) stoichiometric VO_2_, in which the high-tem­per­a­ture phases are metallic (Chamberland, 1973 ▸; Joshi *et al.*, 2023 ▸).

### 3.3. Single-crystal X-ray diffraction to 9.2 GPa at room tem­per­a­ture

**Figure 9:**
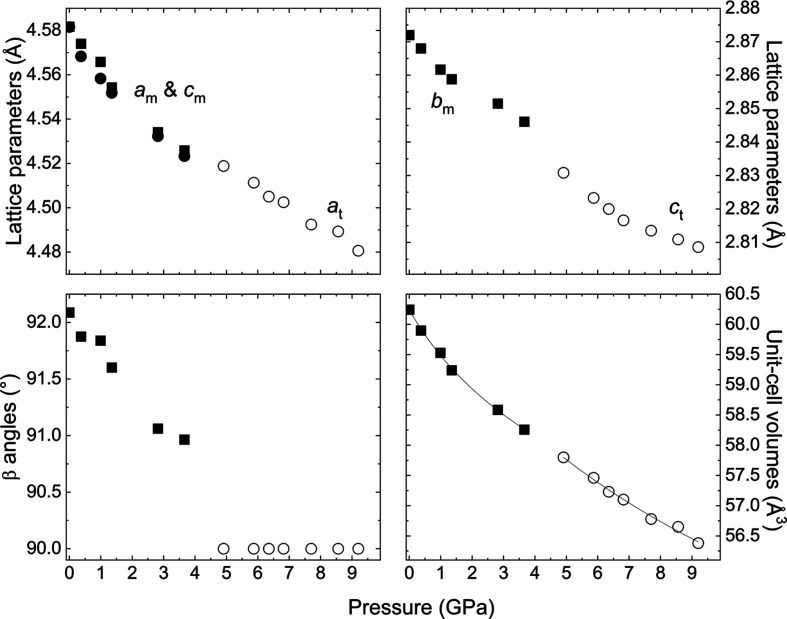
Lattice parameters, β angles and unit-cell volumes for the monoclinic (m, full symbols) and tetra­gonal (t, open symbols) phases on com­pression at room tem­per­a­ture. The estimated standard deviations are smaller than the size of the symbols. The solid lines are the fits of the third-order Murnaghan equations of state.

The high-pressure single-crystal data measured on both laboratory diffractometers up to 9.2 GPa at room tem­per­a­ture were analyzed in the average structure in *P*2/*m* (*Z* = 2). The β angle decreases on com­pression, and it becomes equal to 90° at 4.9 GPa (Fig. 9 ▸). All the reflections at this and higher pressures can be indexed with the tetra­gonal lattice: *a* ≃ 4.5 Å and *c* ≃ 2.8 Å. There is also a discontinuity in the lattice parameters *b*_m_ ↔ *c*_t_, which are determined by the V—V distances in the octa­hedral chains. This implies a first-order phase transition from the monoclinic to tetra­gonal phases at about 3.7–4.9 GPa. The data measured at 6.35 GPa and room tem­per­a­ture on the 4-circle diffractometer could be integrated and refined with the ideal rutile structure (Tables S4–S6). The *P*2/*m* ↔ *P*4_2_/*mnm* phase transition is reversible on decom­pression. All these observations agree with our previous observations about the fact that, on quenching to ambient conditions, rutile V_0.92_O_2_ transforms to a range of its distorted variants depending on the actual highest pressure and tem­per­a­ture reached during the synthesis (Grzechnik *et al.*, 2024 ▸). The behaviour of metal-deficient V_0.92_O_2_ at high pressures and room tem­per­a­ture is therefore different from that of the *M*1 phase of stoichiometric VO_2_, which transforms to a series of low-symmetry polymorphs but not to ideal rutile (Chen *et al.*, 2017 ▸; Bouvier *et al.*, 2023 ▸).

The monoclinic phase is much more com­pressible than the tetra­gonal one. The *P*–*V* data up to 3.66 GPa can be fitted with the third-order Murnaghan equation of state (EoS): *V*_0_ = 60.24 (2) Å^3^, *B*_0_ = 66 (3) GPa and *B*_0_′ = 29 (3). Since the zero-pressure volume *V*_0_ for the tetra­gonal polymorph cannot be determined from the EoS fit, a modified third-order Murnaghan EoS in terms of (*P* – *P*_tr_), where *P*_tr_ = 4.91 GPa in the transition pressure, was used. Consequently, *V*_tr_ = 57.80 (2) Å^3^, *B*_tr_ = 143 (13) GPa and *B*_tr_′ = 16 (8) are obtained at *P*_tr_ = 4.91 GPa. The *B*_0_/*B*_tr_ and *B*_0_′/*B*_tr_′ parameters can be com­pared with those for the different polymorphs of stoichiometric VO_2_. The bulk modulus *B*_0_ for the phase *M*1 determined theoretically (Dong & Liu, 2013 ▸) is 237 GPa, while the reported experimental values are 213 (2) (Bai *et al.*, 2015 ▸) and 194 (7) GPa (Bouvier *et al.*, 2023 ▸). The theoretical *B*_0_ for *M*2 is 241 GPa (Dong & Liu, 2013 ▸). According to the calculations by Dong & Liu (2013 ▸), *B*_0_ for rutile VO_2_ is 243 GPa. The experimental *B*_0_ for rutile at 383 K is 190 (2) GPa (Bai *et al.*, 2015 ▸). The most com­pressible polymorph of stoichiometric vanadium dioxide is VO_2_(B), with *B*_0_ = 129 (4) GPa (Wang *et al.*, 2016 ▸), which is com­parable to V_0.92_O_2_. The first derivatives of the bulk moduli for V_0.92_O_2_ are higher than all those for VO_2_, which are in the range 4–7 (Dong & Liu, 2013 ▸; Bai *et al.*, 2015 ▸; Wang *et al.*, 2016 ▸; Bouvier *et al.*, 2023 ▸).

## 4. Conclusions

Structural modulations are usually stabilized at low tem­per­a­tures as a disordered structure transforms into a more ordered one on cooling. Notable exceptions are brownmillerites Ca_2_Fe_2_O_5_ (Krüger *et al.*, 2005 ▸) and Ca_2_Al_2_O_5_ (Lazic *et al.*, 2008 ▸), as well as inter­metallic PdBi (Folkers *et al.*, 2020 ▸). In the brownmillerites at about 1000 K, the modulation arises from the incommensurate sequence of enanti­omorphic left- and right-handed tetra­hedral *B*O_4_ chains (*B* = Fe or Al). In PdBi with the structure related to TlI, the incommensurability on heating to above 473 K originates from the presence of nearly regular TlI-type slabs in a distorted TlI superstructure with Pd–Pd dimers. Folkers *et al.* (2020 ▸) inter­preted the TlI-type slabs as the regions of higher vibrational freedom that are entropically favoured at high tem­per­a­tures. Metastable V_0.92_O_2_, which is investigated in this study, undergoes a phase transition to an incommensurate phase above about 350 K. Our inter­pretation is that such behaviour is a consequence of an incommensurate way of disordering of V^4+^ and V^5+^ cations, which are chemically and structurally distinct with different ionic radii, in a rigid hexa­gonal close-packing oxygen sublattice. Eventually, V_0.92_O_2_ starts to decom­pose above 460 K at atmospheric pressure. In other words, decom­position is preceded by incommensurability. Such a phenomenon could possibly be found in other materials, not necessarily metastable and synthesized at high pressures.

V_0.92_O_2_, which contains 35 wt% of V_2_O_5_, is insulating. It demonstrates the capacity of the rutile-type framework to accommodate a wide series of VO_2_–V_2_O_5_ com­positions. It also suggests that by varying stoichiometries and pressure–tem­per­a­ture conditions one would synthesize rutile-related V_1–*x*_O_2_ materials with transport properties ranging from metallic to insulating. Since the com­pound with 10 wt% of V_2_O_5_ (V_0.976_O_2_) is indeed metallic (Chamberland, 1973 ▸), it remains to be seen for which higher V_2_O_5_ contents V_1–*x*_O_2_ oxides become insulating. In addition, 35 wt% of V_2_O_5_ does not need to be a com­positional limit for the stability of the rutile-related structure that has one important feature common to all the known (non-)stochiometric VO_2_ phases: the hexa­gonal close-packing oxygen sublattice is rigid, while the cation sublattice is flexible, allowing for various schemes of cation (dis)order. Therefore, incommensurate phases could also be expected for other V_1–*x*_O_2_ com­positions, apart from V_0.92_O_2_.

Metal-deficient V_0.92_O_2_ reversibly transforms to the ideal rutile structure (*P*4_2_/*mnm*, *Z* = 2) at about 5 GPa and room tem­per­a­ture. Altogether, the results of this work demonstrate that its structural behaviour under extreme conditions is distinctly different from that of stoichiometric VO_2_.

Our findings imply then that the structural and electronic (in)stabilities of the non-stoichiometric vanadium dioxides warrant detailed investigations since the occurrence of the metallic *R* phases (*P*4_2_/*mnm*, *Z* = 2) in the mixed-valence VO_2±*x*_ materials for different *x* ≠ 0 is not certain. As our work was focused on Bragg diffraction, further insight into the mechanism of the unusual phase transition observed in V_0.92_O_2_ on increasing the tem­per­a­ture could be obtained by a future detailed investigation of the diffuse scattering, which seems to occur as a precursor effect to the formation of the modulated structure.

## Supplementary Material

Crystal structure: contains datablock(s) global, V0.92O2tetragonal, V0.92O2modulated. DOI: 10.1107/S2052252525010693/zx5035sup1.cif

Structure factors: contains datablock(s) V0.92O2tetragonal. DOI: 10.1107/S2052252525010693/zx5035V0.92O2tetragonalsup2.hkl

Structure factors: contains datablock(s) V0.92O2modulated. DOI: 10.1107/S2052252525010693/zx5035V0.92O2modulatedsup3.hkl

Tables S1-S6 and Fig. S1. DOI: 10.1107/S2052252525010693/zx5035sup4.pdf

Animation S1. DOI: 10.1107/S2052252525010693/zx5035sup5.gif

Animation S2. DOI: 10.1107/S2052252525010693/zx5035sup6.gif

Animation S3. DOI: 10.1107/S2052252525010693/zx5035sup7.gif

Animation S4. DOI: 10.1107/S2052252525010693/zx5035sup8.gif

Animation S5. DOI: 10.1107/S2052252525010693/zx5035sup9.gif

Animation S6. DOI: 10.1107/S2052252525010693/zx5035sup10.gif

tYecoNslA2B

CCDC reference: 2512153

## Figures and Tables

**Figure 6:**
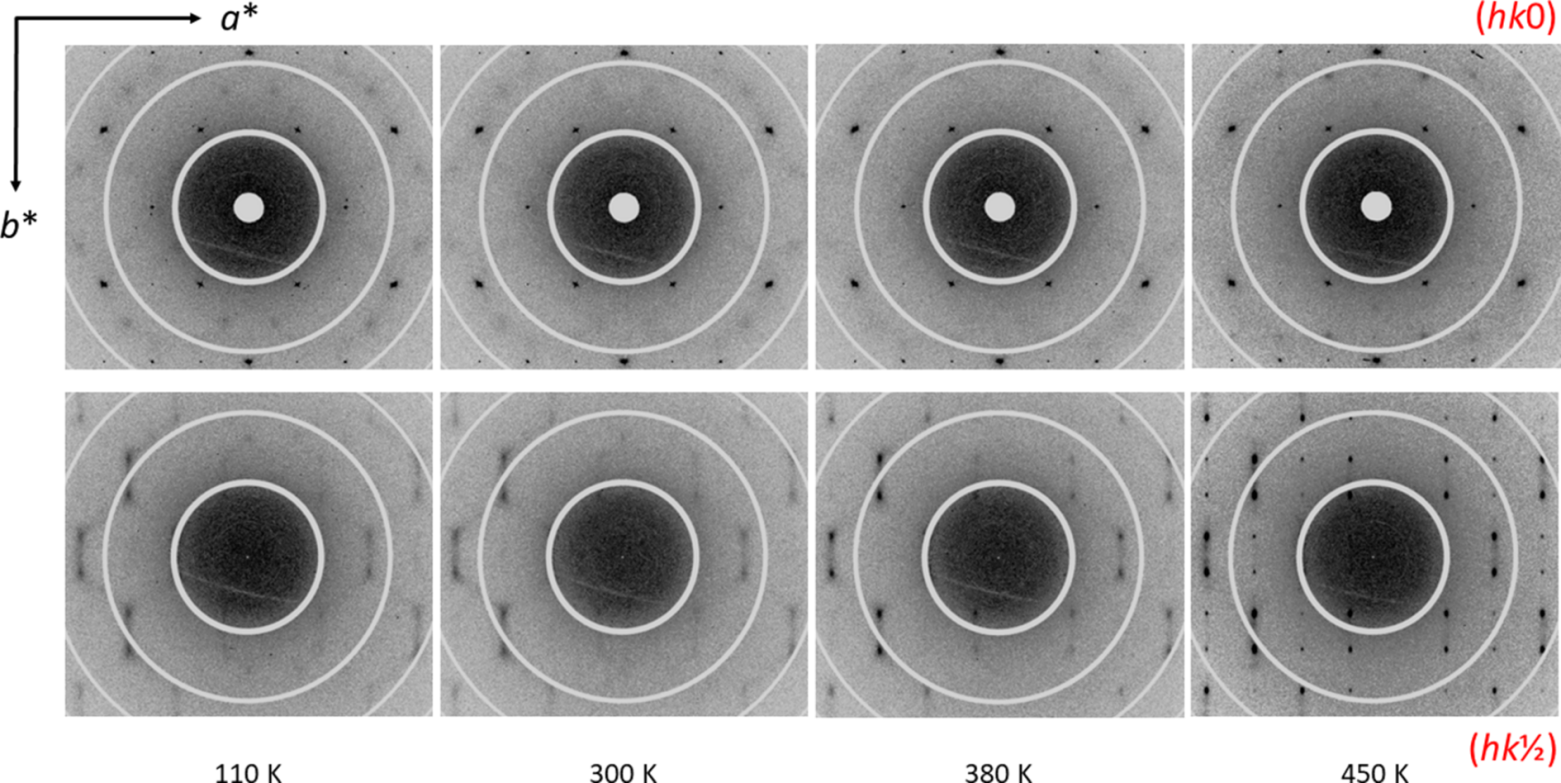
Reconstructions of the reciprocal space in the (*hk*0) and (*hk*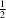) planes (the upper and lower row, respectively) from the synchrotron data at selected tem­per­a­tures.

**Table 1:** Experimental and refinement details at 460 K (λ = 0.72044 Å)

Space group	*X*2/*m*(0β0)*s*0
Centring	*X* = 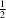 , 0, 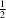 , 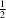
*Z*	4
*a* (Å)	6.6458 (6)
*b* (Å)	2.8746 (2)
*c* (Å)	6.3631 (6)
β (°)	90.024 (10)
*V* (Å^3^)	121.562 (18)
**q** vector	0, 0.2328 (9), 0
ρ (g cm^−3^)	4.3091
μ (mm^−1^)	6.934
	
No. measured reflns	1297
Range of *hkl*	−8 ≤ *h* ≤ 8
	−4 ≤ *k* ≤ 4
	−8 ≤ *l* ≤ 84
	−1 ≤ *m* ≤ 1
θ (min/max)	3.53/32.52
No. symmetry independent reflns (all)	445
No. symmetry independent reflns (obs)*^*a*^*	302
*R*_int_(obs/all)	0.75/0.76
	
All reflns *R*(obs)/*wR*(all)*^*b*^*	5.60/7.28
Main reflns *R*(obs)/*wR*(all)	5.08/6.44
Satellite reflns *R*(obs)/*wR*(all)	9.44/11.74
GoF (obs/all)	5.71/4.44
Twin law	(−100, 0−10, 001)
Twin volumes I/II	0.86 (4)/0.14 (4)
Δρ_max_, Δρ_min_ (e Å^−3^)	0.72, −0.79
No. parameters	92

Symmetry operations: (1) *x*_1_, *x*_2_, *x*_3_, *x*_4_; (2) −*x*_1_, *x*_2_, −*x*_3_, *x*_4_ + 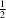; (3) −*x*_1_, −*x*_2_, −*x*_3_, −*x*_4_; (4) *x*_1_, −*x*_2_, *x*_3_, −*x*_4_ + 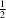. Notes: (*a*) criterion for the observed reflections is |*F*(obs)| > 3σ; (*b*) all agreement factors are given in percent (%) and the weighing scheme is 1/{σ^2^*F*(obs) + [0.01*F*(obs)]^2^}.

**Table 2:** Positional and isotropic displacement parameters of the atoms, as well as Fourier coefficients of the modulation amplitudes at 460 K

	Occupancy	*x*	*y*	*z*	*U*_iso_	
V1	0.492 (4)	0.0	0.0	0.0	0.0318 (11)	
V2	0.428 (4)	0.0	0.5	0.5	0.0283 (13)	
O1	0.5	−0.2003 (2)	0.0	0.5002 (4)	0.0155 (7)	
O2	0.5	0.0005 (3)	0.5	0.2000 (2)	0.0128 (6)	
						
	*x*sin1	*y*sin1	*z*sin1	*x*cos1	*y*cos1	*z*cos1
V1	0.0065 (4)	0.0	0.0003 (6)	0.0	0.0	0.0
V2	−0.0059 (4)	0.0	0.0001 (6)	0.0	0.0	0.0
O1	−0.0004 (3)	0.0	−0.0004 (6)	0.0	−0.0039 (8)	0.0
O2	0.0008 (3)	0.0	0.0001 (5)	0.0	0.0003 (10)	0.0
						
	*u*_11_	*u*_22_	*u*_33_	*u*_12_	*u*_13_	*u*_23_
V1	0.0693 (14)	0.0155 (18)	0.010 (2)	0.0	0.0068 (16)	0.0
V2	0.051 (2)	0.027 (2)	0.007 (3)	0.0	0.0025 (18)	0.0
O1	0.0147 (11)	0.0171 (9)	0.0147 (14)	0.0	0.0009 (12)	0.0
O2	0.0186 (13)	0.0112 (8)	0.0085 (12)	0.0	0.0059 (16)	0.0
						
V1/V2	*u_ij_*sin1 = 0.0					
						
	*u*_11_sin1	*u*_22_sin1	*u*_33_sin1	*u*_12_sin1	*u*_13_sin1	*u*_23_sin1
O1	−0.0009 (11)	−0.0002 (10)	−0.0004 (13)	0.0	0.0012 (15)	0.0
O2	0.0006 (17)	−0.0001 (13)	0.0020 (17)	0.0	−0.0004 (9)	0.0
	*u*_11_cos1	*u*_22_cos1	*u*_33_cos1	*u*_12_cos1	*u*_13_cos1	*u*_23_cos1
						
V1	For *i* = *ju_ij_*cos1 = 0.0			0.0007 (9)	0.0	0.0012 (14)
V2				−0.0001 (11)	0.0	−0.0004 (16)
O1				0.0012 (7)	0.0	−0.0024 (15)
O2				−0.0008 (7)	0.0.	−0.0005 (12)
						
	*D*_1111_	*D*_1113_	*D*_1122_	*D*_1133_	*D*_1223_	*D*_1333_
V1	−0.0975 (13)	0.0019 (12)	−0.006 (3)	−0.0009 (8)	−0.005 (3)	−0.0007 (19)
V2	−0.019 (2)	−0.0022 (18)	−0.017 (3)	0.0017 (12)	−0.007 (3)	−0.003 (2)
						
	*D*_2222_	*D*_2233_	*D*_3333_	*D*_1112_ = *D*_1123_ = *D*_1222_ = *D*_1233_ = *D*_2223_ = *D*_2333_ = 0.0		
V1	0.07 (4)	−0.009 (3)	0.000 (3)			
V2	−0.10 (4)	−0.012 (3)	−0.002 (3)			
						
	*C*_111_sin1	*C*_113_sin1	*C*_122_sin1	*C*_133_sin1	*C*_223_sin1	*C*_333_sin1
V1	−0.048 (2)	0.006 (2)	−0.015 (4)	−0.0037 (13)	−0.000 (5)	0.002 (3)
V2	0.022 (3)	−0.004 (2)	0.016 (5)	0.0020 (12)	0.000 (6)	0.000 (4)
						
V1/V2	*C*_112_sin1 = *C*_123_sin1 = *C*_222_sin1 = *C*_233_sin1 = 0.0					
V1/V2	*C_ijk_*cos1 = 0.0					
						
	*D*_1112_cos1	*D*_1123_cos1	*D*_1222_cos1	*D*_1233_cos1	*D*_2223_cos1	*D*_2333_cos1
V1	−0.000 (3)	0.002 (2)	0.008 (9)	−0.0002 (12)	0.007 (11)	−0.000 (4)
V2	0.006 (3)	−0.002 (3)	−0.004 (10)	0.0004 (15)	0.005 (13)	−0.002 (4)
						
V1/V2	*D*_1111_cos1 = *D*_1113_cos1 = *D*_1122_cos1 = *D*_1133_cos1 = *D*_1223_cos1 = *D*_1333_cos1 = *D*_2222_cos1 = *D*_2233_cos1 = *D*_3333_cos1 = 0.0					
V1/V2	*D_ijkl_*sin1 = 0.0					

## Data Availability

The data supporting the results reported here can be accessed within the article and supporting information.

## Footnotes

1 The incommensurate vector would be **q** = (0, 0, γ), where γ ≈ 0.234, in the pseudo-ortho­rhom­bic *C*-centred cell derived from the primitive monoclinic one using the transformation *a*′ = *a* + *c*, *b*′ = −*a* + *c* and *c*′ = −*b*.

2 It is noteworthy that part of the elements of the anharmonic tensor and the coefficients of the modulation function of the anharmonic tensor elements refined to values below 3σ. If one restricts these parameters to 0, the overall number of parameters is halved (approximately 40). At the same time, the agreement factors for the satellite reflections increase by approximately 2% [*R*(obs) = 11.54% and w*R*(all) = 13.84%].
