# Supplementary material for: Phase transitions in rutile-related V0.92O2 synthesized at high pressures and tem­per­a­tures
Source: IUCrJ. 2026 Jan 1;13(Pt 1):116–25. doi: 10.1107/S2052252525010693 (PMC12809505; doi:10.1107/S2052252525010693)
Supplement: Supplementary file 4 [file m-13-00116-sup4.pdf]

IUCrJ

Volume 13 (2026)

Supporting information for article:

Phase transitions in rutile-related $V_{0.92}O_2$ synthesized at high pressures and temperatures

Andrzej Grzechnik, Václav Petříček, Pascal Reiss, Paul Zakalek, Dmitry Chernyshov and Karen Friese

Table S1 O-O distances up to 3 Å in the modulated structure at 460 K.

	average	minimum	maximum
$O1-O1^{i}$	2.8746(4)	2.860(2)	2.890(2)
$O1-O1^{ii}$	2.8746(4)	2.860(2)	2.890(2)
$O1-O1^{iii}$	2.663(3)	2.663(4)	2.663(4)
O1-O2	2.739(2)	2.731(4)	2.746(4)
$O1-O2^{i}$	2.739(2)	2.731(4)	2.746(4)
$O1-O2^{iv}$	2.763(2)	2.755(4)	2.772(4)
$O1-O2^{v}$	2.763(2)	2.755(4)	2.772(4)
$O1-O2^{vi}$	2.733(2)	2.723(4)	2.742(4)
$O1-O2^{vii}$	2.733(2)	2.723(4)	2.742(4)
$O1-O2^{viii}$	2.769(2)	2.760(4)	2.778(4)
$O1-O2^{ix}$	2.769(2)	2.760(4)	2.778(4)
$O2-O2^{i}$	2.8746(4)	2.873(3)	2.876(3)
$O2-O2^{ii}$	2.8746(4)	2.873(3)	2.876(3)
$O2-O2^{x}$	2.545(2)	2.545(5)	2.545(5)

Symmetry code: (i) x,y-1,z; (ii)x, y+1,z; (iii)-x, y,-z+1; (iv) x-1/2,y-1,z+1/2; (v) x-1/2,y,z+1/2; (vi)-x,y-1,-z+1; (vii) -x,y,-z+1; (viii) –x-1/2,y-1,-z+1/2; (ix) –x-1/2,y,-z+1/2; (x) –x,y,-z

Table S2 V-V distances (in Å) up to 3.5 Å in the modulated structure at 460 K.

	average	minimum	maximum
$V1-V1^{i}$	2.87489(3)	2.87460(4)	2.87518(4)
$V1-V1^{ii}$	2.87489(3)	2.87460(4)	2.87518(4)
V1-V2	3.492(3)	3.490(4)	3.493(4)
$V1-V2^{iii}$	3.492(3)	3.490(4)	3.493(4)
$V1-V2^{iv}$	3.492(3)	3.490(4)	3.493(4)
$V2-V2^{i}$	2.87484(3)	2.87460(4)	2.87508(4)
$V2-V2^{ii}$	2.87484(3)	2.87460(4)	2.87508(4)

Symmetry code: (i) x,y-1,z; (ii) x,y+1,z; (iii) x,y-1,z-1; (iv) x,y-1,z;

Table S3 V-O distances (in Å) in the modulated structure at 460 K.

	average	minimum	maximum
V1-O1^{i}	1.992(2)	1.951(4)	2.032(4)
V1-O1^{ii}	1.992(2)	1.951(4)	2.032(4)
V1-O2	1.920(3)	1.918(3)	1.922(3)
V1-O2 ^{iii}	1.920(3)	1.918(3)	1.922(3)
V1-O2^{iv}	1.920(3)	1.918(3)	1.922(3)
V1-O2^{v}	1.920(3)	1.918(3)	1.922(3)
V2-O1	1.959(2)	1.939(2)	1.980(2)
V2-O1^{vi}	1.959(2)	1.939(2)	1.980(2)
V2-O1^{vii}	1.959(2)	1.939(2)	1.980(2)
V2-O1^{viii}	1.959(2)	1.939(2)	1.980(2)
V2-O2	1.909(3)	1.909(6)	1.910(6)
V2-O2^{vii}	1.909(3)	1.909(6)	1.910(6)

Symmetry code: (i) x+1/2, y, z-1/2; (ii) –x-1/2, y, -z+1/2; (iii) x,y-1,z; (iv) -x,y-1,-z; (v) -x,y,-z; (vi) x,y+1,z; (vii) -x,y,-z+1; (viii) -x,y+1,-z+1;

Table S4 Experimental and refinement details at 6.35 GPa and room temperature (λ = 0.5608 Å).

Space group	$P4_2/mnm$
Z	2
a (Å)	4.505(2)
c (Å)	2.820(1)
V (Å^3)	57.23(4)

ρ (g cm^{-3})	4.58
μ (mm^{-1})	3.73
No. meas. refl.	373
Range of *hkl*	$-5 \leq h \leq 5$
	$-6 \leq k \leq 6$
	$-3 \leq l \leq 3$
θ(min/max)	3.5/24.49
No. obs. refl.[a]	216
R_{int}(obs/all)	10.70/10.93
R(obs/all)[b]	5.97/9.08
*w*R(obs/all)	5.39/5.68
GoF(obs(all)	2.59/2.14
No. pars.	5

----

[a] Criterion for the observed reflections is |F(obs)| > 3*σ*.

[b] All agreement factors are given in %, weighing scheme is $1/[\sigma^2 F(obs) + (0.01 F(obs))^2]$.

Table S5 Positional and displacement parameters of the atoms at 6.35 GPa and room temperature: $P4_2/mnm$ ($Z = 2$), $a = 4.505(2)$ Å, $c = 2.820(1)$ Å, V = 57.23(4) Å^3.

Atom	Occupancy	*x*	*y*	*z*	U_{iso}
V	0.92	0.5	0.5	0	0.028(2)
O	1	0.203(1)	0.203(1)	0	0.009(2)

Table S6 Interatomic distances (in Å) at 6.35 GPa and room temperature: $P4_2/mnm$ ($Z = 2$), $a = 4.505(2)$ Å, $c = 2.820(1)$ Å, V = 57.23(4) Å^3.

V-V^{i}	2.820(3)
V-V^{ii}	2.820(3)
V-O	1.895(5)
V-O^{iii}	1.895(5)
V-O^{iv}	1.912(4)
V-O^{v}	1.912(4)
V-O^{vi}	1.912(4)
V-O^{vii}	1.912(4)
O-O^{i}	2.820(3)
O-O^{ii}	2.820(3)
O-O^{viii}	2.582(7)
O-O^{ix}	2.691(6)
O-O^{x}	2.691(6)
O-O^{iv}	2.691(6)

O-O^{v}	2.691(6)
O-O^{xi}	2.691(6)
O-O^{xii}	2.691(6)
O-O^{vi}	2.691(6)
O-O^{vii}	2.691(6)

Symmetry code: (i) x,y,z-1; (ii) x,y,z+1; (iii) -x+1,-y+1,z;
(iv) -y+1/2,x+1/2,z-1/2; (v) -y+1/2,x+1/2,z+1/2;
(vi) y+1/2,-x+1/2,z-1/2; (vii) y+1/2,-x+1/2,z+1/2;
(viii) -x,-y,z; (ix) -y+1/2,x-1/2,z-1/2; (x) -y+1/2,x-1/2,z+1/2;
(xi) y-1/2,-x+1/2,z-1/2; (xii) y-1/2,-x+1/2,z+1/2

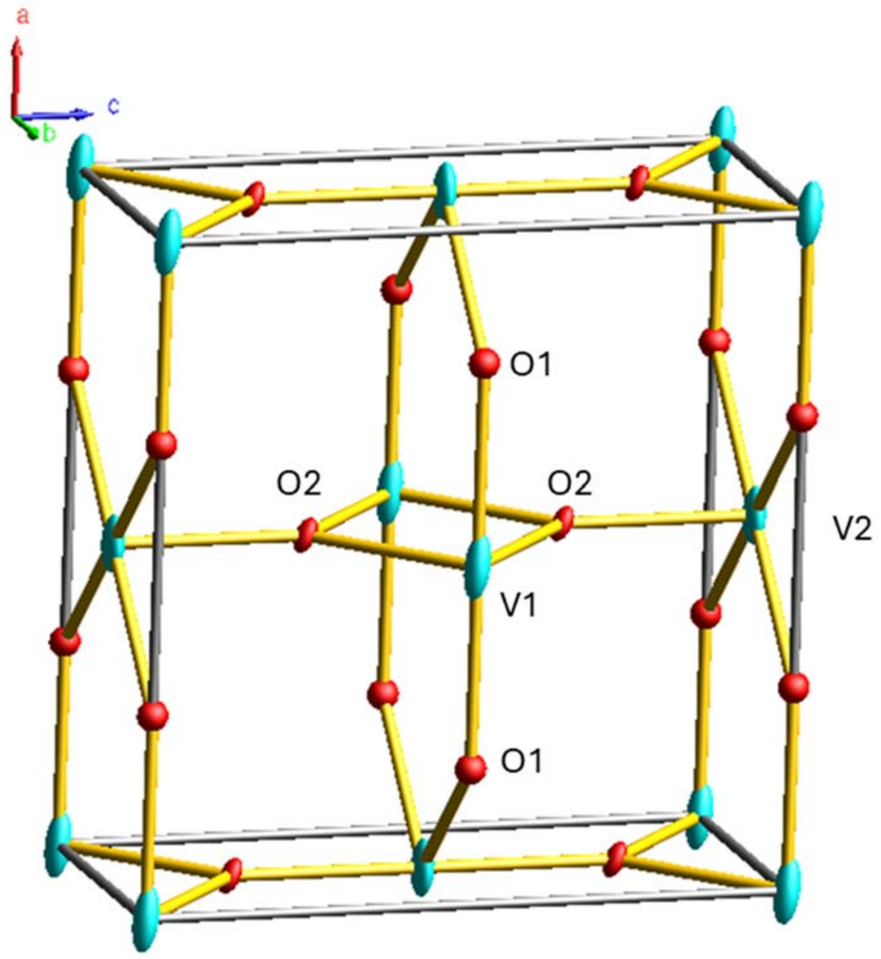

Figure S1 Average structure at 460 K.
