# Supplementary figures and images for: Phase transitions in rutile-related V0.92O2 synthesized at high pressures and tem­per­a­tures
Source: IUCrJ. 2026 Jan 1;13(Pt 1):116–25. doi: 10.1107/S2052252525010693 (PMC12809505; doi:10.1107/S2052252525010693)

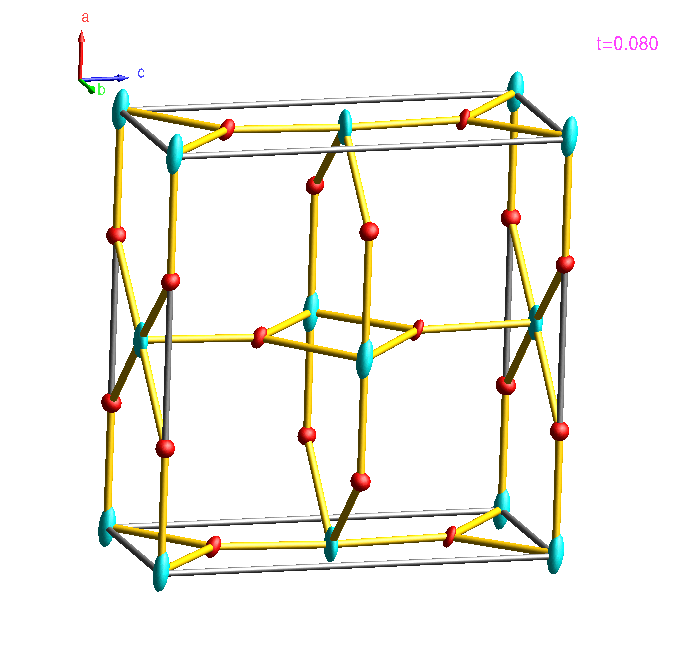

Supplement: Supplementary file 5 [file m-13-00116-sup5.gif]

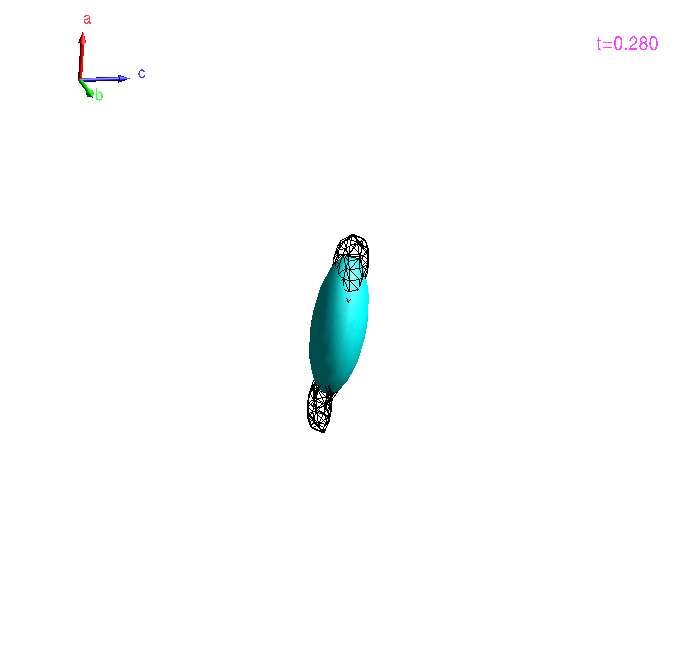

Supplement: Supplementary file 6 [file m-13-00116-sup6.gif]

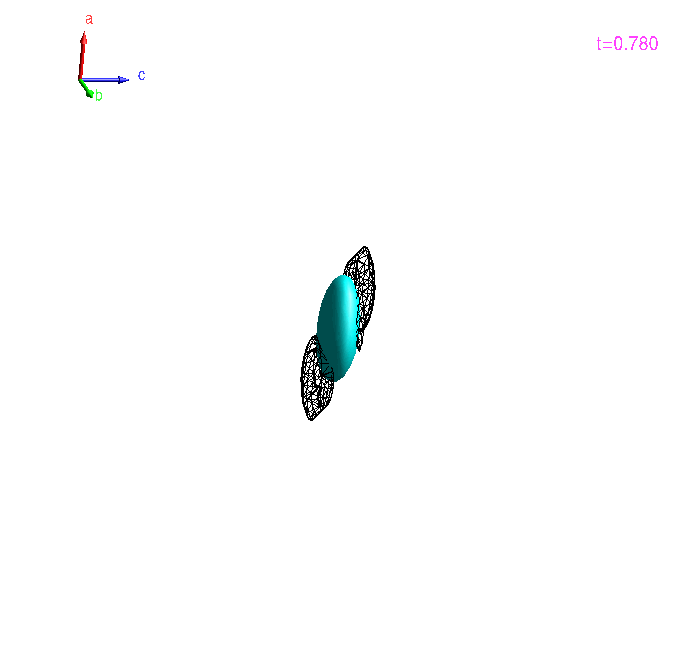

Supplement: Supplementary file 7 [file m-13-00116-sup7.gif]

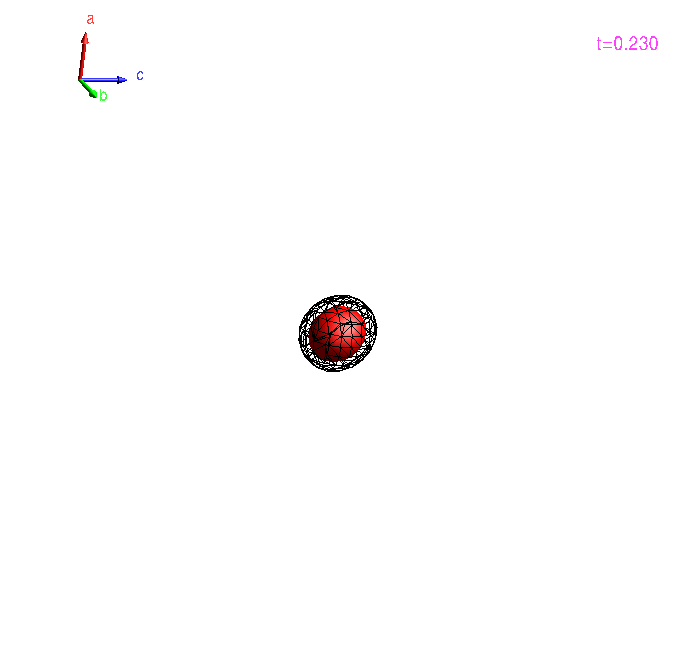

Supplement: Supplementary file 8 [file m-13-00116-sup8.gif]

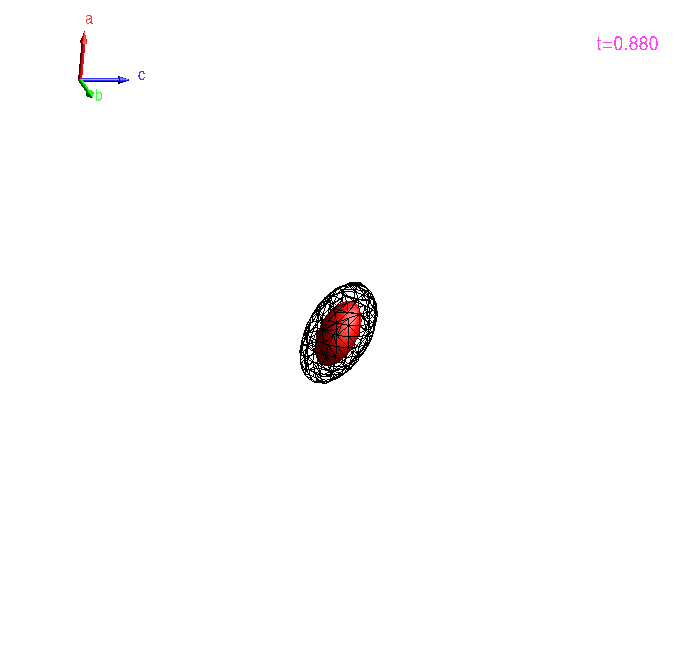

Supplement: Supplementary file 9 [file m-13-00116-sup9.gif]

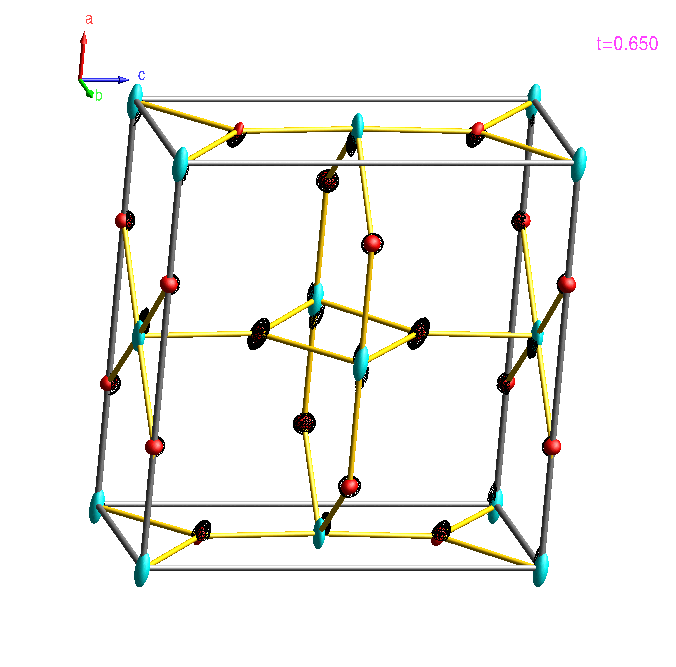

Supplement: Supplementary file 10 [file m-13-00116-sup10.gif]
